# Exosomal microRNA panel as a diagnostic biomarker in patients with hepatocellular carcinoma

**DOI:** 10.3389/fcell.2022.927251

**Published:** 2022-09-23

**Authors:** Jingwen Yang, Weiwei Dong, He Zhang, Huixia Zhao, Zhiyan Zeng, Fengyun Zhang, Qiuwen Li, Xiaohong Duan, Yanyan Hu, Wenhua Xiao

**Affiliations:** ^1^ Senior Department of Oncology, The Fifth Medical Center of PLA General Hospital, Beijing, China; ^2^ Department of Oncology, 4th Medical Center of PLA General Hospital, Beijing, China; ^3^ ChosenMed Technology (Beijing) Co., Ltd., Beijing, China

**Keywords:** exosome, plasma, microRNA, diagnostic biomarker, hepatocellular carcinoma

## Abstract

**Background:** Diagnostic tools for hepatocellular carcinoma (HCC) are critical for patient treatment and prognosis. Thus, this study explored the diagnostic value of the exosomal microRNA panel for HCC.

**Methods:** Expression profiles of microRNAs in exosomes and plasma of HCC and control groups were assessed using microRNA microarray analysis. Reverse transcription-quantitative PCR was applied to evaluate the expression of candidate microRNAs in blood samples from 50 HCC patients, 50 hepatic cirrhosis patients, and 50 healthy subjects. The area calculated the diagnostic accuracy of the microRNAs and microRNA panel under the receiver operating characteristic curve (AUC).

**Results:** MicroRNA microarray analysis revealed that there were more differentially expressed microRNAs in the exosome HCC group than plasma HCC group. Among the 43 differentially expressed microRNAs contained in both exosomes and plasma, we finally decided to testify the expression and diagnostic significance of microRNA-26a, microRNA-29c, and microRNA-199a. The results indicated that expression of the microRNA-26a, microRNA-29c, and microRNA-199a in both exosomes and plasma was significantly lower in HCC patients compared with hepatic cirrhosis and healthy group. Interestingly, exosomal microRNAs were substantially more accurate in diagnosing HCC than microRNAs and alpha-fetoprotein in plasma. Moreover, the exosomal microRNA panel containing microRNA-26a, microRNA-29c, and microRNA-199a showed high accuracy in discriminating HCC from healthy (AUC = 0.994; sensitivity 100%; specificity 96%) and hepatic cirrhosis group (AUC = 0.965; sensitivity 92%; specificity 90%).

**Conclusion:** This study revealed that the exosomal microRNA panel has high accuracy in diagnosing HCC and has important clinical significance.

## Introduction

Worldwide, liver cancer is the second leading cause of cancer death in men and the sixth leading cause of cancer death in women. An estimated 905,677 new liver cancer cases and 830,180 deaths occurred worldwide in 2020 (Sung et al., 2021). Hepatocellular carcinoma (HCC) accounts for 75%–85% of all primary liver cancers. And China is one of the most high-risk HCC areas because of chronic hepatitis B virus infection and aflatoxin exposure. The prognosis of HCC patients has been improved after partial hepatectomy and liver transplantation is performed. However, only 10%–23% of HCC patients are candidates for these surgical interventions because most patients present with advanced HCC at the time of diagnosis (Ayoub et al., 2019). Accordingly, timely and efficient early diagnosis and cancer intervention can effectively improve the prognosis of patients. Currently, the standard methods for evaluating suspicious liver nodules include diagnostic imaging with computed tomography or magnetic resonance imaging and the measurement of serum alpha-fetoprotein (AFP). However, clinical studies have shown that the measurement of AFP does not have adequate sensitivity and specificity for early detection of HCC (Chen et al., 2015). In previous studies, some miRNA or lncRNA identified diagnostic or prognostic biomarkers in HCC or other cancers (Sasaki et al., 2019; Yuan et al., 2021a; Ren et al., 2021; Ren et al., 2022). Tissue biopsy is still needed for accurate diagnosis of HCC, which is invasive and inconvenient to perform. Therefore, exploring efficient and reliable early diagnosis methods for the prognosis of HCC patients is of great significance.

Exosomes are cup-shaped nanovesicles with a diameter of 30–150 µm, released into circulation by multiple cell types, including tumor cells. *t* has been suggested that tumor-derived exosomes quantitatively predominate in the peripheral blood of cancer patients (Challagundla et al., 2014). The use of exosomes in the clinical setting for diagnostic, prognostic, therapeutic, and drug delivery tools has been well demonstrated and remains a topic of intense research based on the ever-growing literature on the subject (Doyle and Wang, 2019). Exosomes’ role, as defined by their intracellular endosomal origin, remains unknown because *in vivo* tools for tracking exosome release and biogenesis are still lacking (Bebelman et al., 2018). Numerous proteins and nucleic acids including microRNAs (miRNAs) are present in exosomes, which are representative of the secreting cells (Zhang et al., 2015). MiRNAs exist naturally as the most biologically stable nucleic acid molecules with about 18–25 nucleotides in length, which can interact with the target genes to suppress their expression by promoting the degradation of target genes or inhibiting translation (Dai et al., 2019). Exosome-mediated miRNA transduction plays a pivotal role in the dialogue between human tumors and their microenvironment (Hannafon and Ding, 2013). Moreover, miRNAs in exosomes have shown extra stability under different storage conditions, which may be attributed to the protection of the lipid membrane of exosomes (Ge et al., 2014). These characteristics make exosomal miRNA become a novel biomarker for the diagnosis and prognosis of numerous diseases, including HCC (Barcelo et al., 2019; Ingenito et al., 2019; Ji et al., 2019; Lin and Zhang, 2019; Min et al., 2019). In addition, an increasing number of studies have revealed that miRNA panel is more accurate than single miRNAs in diagnosis of the disease and prediction of the prognosis (Elemeery et al., 2017; Elemeery et al., 2019; Ning et al., 2019; Wang et al., 2020). These findings provoked us to explore whether exosome miRNA can improve the prognosis of patients.

In the current study, we aimed to investigate the diagnostic role of exosomal miRNAs in HCC and provided three diagnostic biomarkers of microRNA-26a, microRNA-29c, and microRNA-199a.

## Materials and methods

### Patients and samples

Peripheral blood samples from 50 patients with HCC were obtained at the Chinese PLA General Hospital (Beijing, China) from September 2013 to November 2014. The inclusion criteria used for the enrolment of patients were: age between 18 and 80 years, confirmed diagnosis of HCC by histological examination, under palliative therapy or without any treatment, and the absence of other malignant tumors. Meanwhile, blood samples were also collected from 50 hepatic cirrhosis patients and 50 healthy subjects matching age and gender to the HCC patients in the same period. All the plasma samples were collected, centrifuged at 2,000 g for 5 min at room temperature, and stored as 500 μl aliquots at −80°C before experimental use. We also downloaded the miRNAs expression data of liver hepatocellular carcinoma (LIHC) from The Cancer Genome Atlas (TCGA) (http://xena.ucsc.edu/). We used the *R* package ‘SVA (Leek et al., 2012) to remove batch effects, with “combat” function to normalize the miRNA expression in TCGA and our datasets.

This study was conducted under the World Medical Association’s Declaration of Helsinki for experiments involving humans. All experimental protocols were approved by the Ethics Committee of the Fifth Medical Center of Chinese PLA General Hospital and informed written consent was obtained from each participant prior to the recruitment.

### Isolation of exosomes from peripheral blood

Exosomes in plasma were extracted using ExoQuick Exosome Precipitation Solution (System Biosciences, Palo Alto, CA, United States) per the manufacturer’s instructions. To remove cells and cell debris, blood samples were centrifuged at 3,000 g for 15 min. The supernatant was transferred to a new tube and added with ExoQuick Exosome Precipitation Solution. After incubation at 4°C for 30 min, the mixture was centrifuged at 1,500 g for 30 min. The supernatant was discarded and the precipitated exosomes were centrifuged again at 1,500 g for 5 min to remove all fluid traces. The exosome pellet was resuspended in 200 μl PBS and was used immediately or stored at −80°C.

### RNA isolation and quantitative real-time PCR

Total RNA in plasma or exosomes was extracted using the Total Exosome RNA and Protein Isolation Kit (Thermo Fisher Scientific, Waltham, MA, United States) according to the manufacturer’s instructions. The exosome solution or plasma was added to Denaturing Solution and incubated on ice for 5 min. Then the mixture was added with Acid-Phenol: Chloroform and mixed thoroughly. Centrifugation was performed at 10,000 g for 5 min and the aqueous phase was transferred to a new tube. 100% ethanol was added to the aqueous phase and the mixture was transferred onto the filter cartridge. Centrifugation was performed at 10,000 g for 15 s and the flow-through was discarded. Wash Solution 1 and 2/3 were added to the filter cartridge in turn and centrifugation was performed each time. The residual fluid was removed from the filter by centrifugation. Then the filter cartridge was transferred into a new collection tube and a 50 μl preheated Elution Solution was applied to recover the RNA. The extracted RNA was quantified with NanoDrop ND-1000 (Thermo Fisher Scientific) and stored at −80°C.

qRT-PCR was performed to detect the expression of miRNAs in exosomes and plasma. Total RNA was transformed into cDNA using Taqman MicroRNA Reverse Transcription Kit (Thermo Fisher Scientific), according to the manufacturer’s instructions. Quantitative PCR was then performed using Taqman microRNA assays on ABI Prism 7900HT Detection System (Thermo Fisher Scientific), as per the manufacturer’s instructions. The PCR cycling conditions were as follows: 1) 50°C for 2 min; 2) 95°C for 10 min; 3) 40 cycles of 95°C for 15 s, 60°C for 1 min. The primers and probes were designed and produced by Thermo Fisher Scientific: U6 snRNA (assay ID 001973), has-miR-29c (assay ID 000587), has-miR-26a (assay ID 000405), and has-miR-199a (assay ID 000498). U6 was used as a stable endogenous control for normalization. The relative gene expression was calculated as the value of 2^−ΔCq^. ΔCq was defined as the difference in Cq value between specific miRNA and U6. All assays were carried out in triplicate. The expression of miRNA data was show in Supplementary Table S1.

### Observation of exosomes by transmission electron microscopy

Exosome suspension was deposited on a copper grid for 5 min at room temperature. Then the exosomes were stained with 2% uranyl acetate solution for 1 min and dried for 20 min at room temperature. The samples were observed and photographed under transmission electron microscopy (Tecnai G2 Spirit BioTwin, FEI, Hillsboro, OR, United States).

### Identification of exosomes by nanoparticle tracking analysis

Exosome samples resuspended in particle-free PBS were diluted 200 times and examined using ZetaVIEW S/N 17-310 (Particle Metrix, Meerbusch, Germany) equipped with a 405 nm laser. And the size distribution and concentration of the particles were analyzed by ZetaView (Version 8.04.02).

### Analysis of exosomes by western blot

Exosomes were lysed in cold RIPA buffer with PMSF. The total protein concentration was determined by BCA assay. Protein samples were mixed with 5× loading buffer and separated by 10% SDS polyacrylamide gels. Then the proteins were transferred to PVDF membranes (Millipore, NJ, United States) and incubated with 5% non-fat milk at room temperature for 1 h. Exosomes were identified using 2 positive markers including CD9 (1:1,000; EXOAB-CD9A-1, System Biosciences) and CD63 (1:1,000; EXOAB-CD63A-1, System Biosciences). Calnexin (1:1,000; YT0613, Immunoway Biotechnology) was used as a negative marker for exosomes.

### MicroRNAs microarray analysis

The miRNA 4.0 Array (Affymetrix, Santa Clara, CA, United States), which contained 30,424 (including rodent) miRNAs from the latest miRBase database, was used to detect the expression profiles of miRNAs in exosomes and plasma. The FlashTag Biotin HSR RNA Labeling Kit was used to label the RNAs, after which ELOSA QC Assay was performed. Then the RNAs were hybridized with the Affymetrix GeneChip 645 System and washed with Affymetrix GeneChip 450 System. The hybridized sequences were observed using the Affymetrix GeneChip 7G Microarray Scanner. Scanned images were imported into Affymetrix Expression Console Software for grid alignment and expression data analysis. A Fold Change filtering was performed to identify differentially expressed genes, the threshold is Fold Change ≥2.0 or ≤ −2.0. A value of *p* < 0.05 was considered significantly different. The miRNA array data were show in Supplementary Table S2.

### Functional enrichment analysis

Han et al. (2016) summarized 1722 miRNAs’ 85,449 targets from four public databases (miRTarBase, TarBase, miRecords, and mir2Disease). We selected the miRNA targets of miR-26a, miR-29c, and miR-199a for functional enrichment analysis. Kyoto Encyclopedia of Genesand Genomes (KEGG) pathway enrichment analysis were performed for three miRNAs by using the online tool (http://vip.sangerbox.com/) (Kanehisa and Goto, 2000; Kanehisa, 2019; Kanehisa et al., 2021). KEGG pathways with *p*-values < 0.05 were statistically significant.

### Statistical analysis

All data were presented as mean ± standard deviation or *n* (%). The characteristics of the participants and relative expression of the miRNAs were compared among the HCC group (*n* = 50), hepatic cirrhosis group (*n* = 50), and healthy group (*n* = 50). Numerical data were analyzed by the Shapiro-Wilk test for assessment of normality and the comparison among different groups was carried out by one-way analysis of variance or the Kruskal-Wallis test. Categorical data were calculated by the Chi-square test or Fisher’s exact test. In addition, the predicted probability of being diagnosed with HCC was used as a surrogate marker to construct the receiver operating characteristic (ROC) curve (Yuan et al., 2021b). And the area under the curve (AUC) was used as an accuracy index to evaluate the diagnostic performance of the miRNAs, miRNA panel, or AFP. Data from RT-qPCR were statistically analyzed using SPSS software (Version 18.0, SPSS, Chicago, IL, United States) and the ROC curve analysis was computed using MedCalc software (Version 11.0.3.0, MedCalc software, Mariakerke, Belgium). A value of *p* < 0.05 was considered significantly different.

## Results

### Patient characteristics

A total of 150 participants, including 50 HCC patients, 50 hepatic cirrhosis patients, and 50 healthy subjects, were recruited to detect miRNAs in exosomes and plasma. The characteristics of these participants are presented in Table 1. All the HCC patients were diagnosed with liver cirrhosis. There were no differences in age, gender, and laboratory results such as total bilirubin (TBL) and albumin (ALB) among the healthy, hepatic cirrhosis and HCC group. Laboratory results such as hepatitis B surface antigen (HBsAg) and hepatitis B e antigen (HBeAg) were similar between hepatic cirrhosis and the HCC group. However, the mean value of alanine aminotransferase (ALT) was 92.4 U/L in the HCC group, 48.4 U/L in Hepatic cirrhosis group, and 24.7 U/L in the Healthy group. The ALT was significantly higher in the HCC group than in the other two groups (*p* < 0.001). Meanwhile, the HCC group had a higher AFP value than the hepatic cirrhosis group (*p* = 0.012).

**TABLE 1:** Characteristics of the participants enrolled in the study.

Variable	Healthy group (*n* = 50)	Hepatic cirrhosis group (*n* = 50)	HCC group (*n* = 50)	*p*-value
Age (years)	61.5 ± 5.2	63.0 ± 3.4	60.8 ± 5.3	0.556^a^
Gender				
Male	26 (52)	29 (58)	30 (60)	
Female	24 (48)	21 (42)	20 (40)	0.667^a^
TBL (μmol/l)	15.5 ± 2.3	13.1 ± 4.3	11.1 ± 8.2	0.325^a^
ALB (g/dl)	40.2 ± 6.6	38.7 ± 4.5	39.4 ± 5.7	0.413^a^
ALT (U/L)	24.7 ± 11.1	48.4 ± 20.2	92.4 ± 57.5	<0.001^a^
AFP (ng/ml)				
≤400	50 (100)	46 (92)	29 (58)	
>400	0 (0)	4 (8)	21 (42)	0.012^b^
HBsAg				
Positive	0 (0)	42 (84)	39 (86)	
Negative	50 (100)	8 (16)	11 (14)	0.086^b^
HBeAg				
Positive	0 (0)	30 (60)	35 (70)	
Negative	50 (100)	20 (40)	15 (30)	0.171^b^
Liver cirrhosis				
Yes	0 (0)	50 (100)	50 (100)	
No	50 (100)	0 (0)	0 (0)	

a the comparison was among healthy, hepatic cirrhosis and HCC group.

b the comparison was between hepatic cirrhosis and HCC group.

### Identification of exosomes derived from plasma of hepatocellular carcinoma patients

**FIGURE 1:**
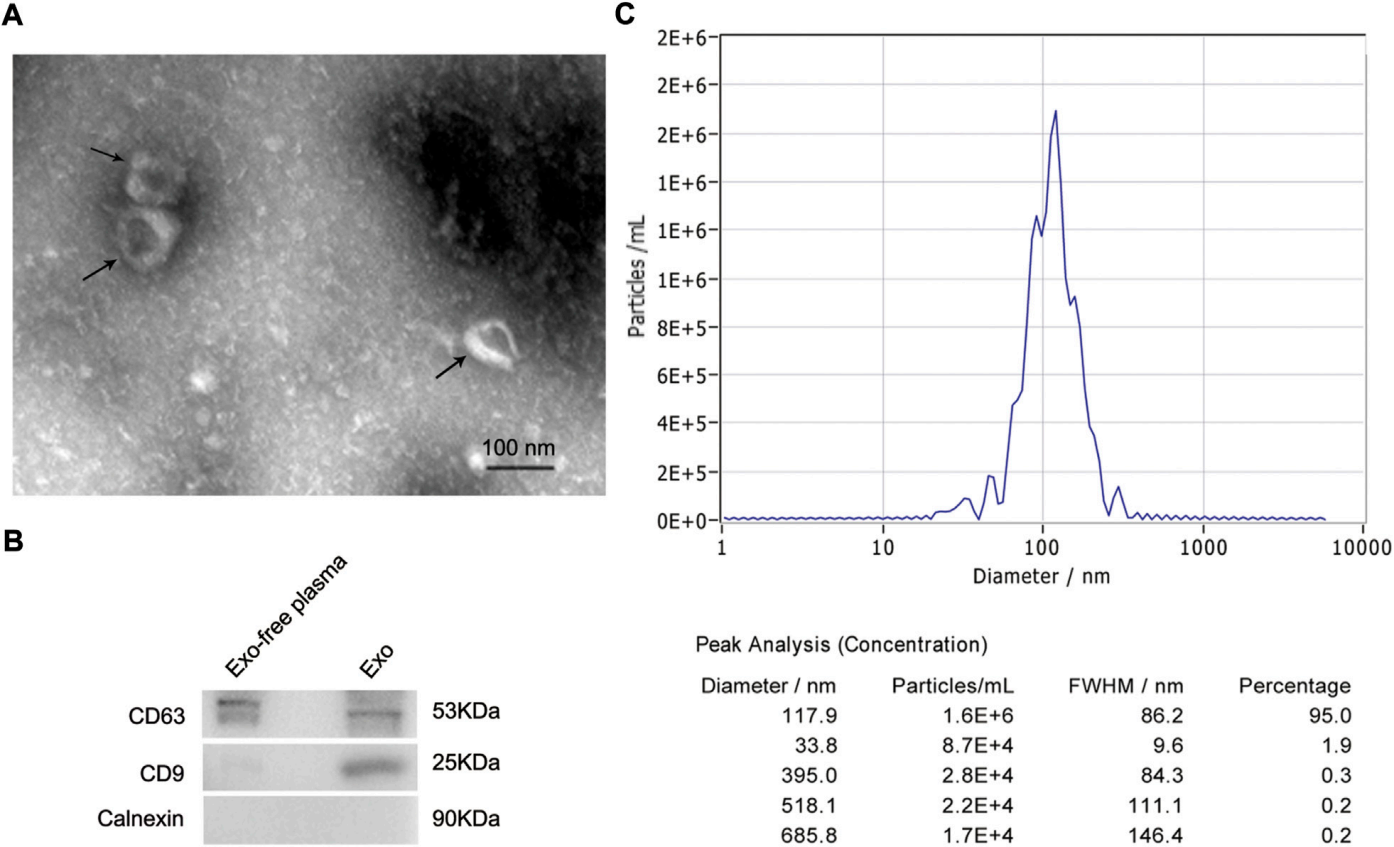
Identification of exosomes derived from plasma of HCC patients. **(A)** Representative TEM images of exosomes isolated from plasma of HCC patients. Black arrows indicating typical exosomes. Bar = 100 nm. **(B)** Exosome-enriched protein markers, including CD63 (53 kDa) and CD9 (25 kDa), and a negative marker, Calnexin (90 kDa), were analyzed by western blot. The exosome-depleted plasma was used as a control. **(C)** Size distribution of exosomes ranged from 50 to 150 nm as analyzed by nanoparticle tracking analysis. HCC, hepatocellular carcinoma; TEM, transmission electron microscope.

TEM, NTA, and western blot identified exosomes derived from plasma. TEM results confirmed the presence of exosomes, which were cup-shaped vesicles with bilayer (Figure 1A). And the purity of exosomes was estimated by western blot. Positive protein markers, including CD9 and CD63, were both detected in isolated exosomes, and the negative marker Calnexin was absent (Figure 1B). In addition, NTA analysis demonstrated that the peak size of most exosomes (95%) was 117.9 nm and the median size was 109.5 nm, which was consistent with results from other researchers (Figure 1C).

### MicroRNA screening by microarray analysis

This study used plasma and exosome solutions derived from 3 HCC patients, 3 hepatic cirrhotic patients, and 3 healthy volunteers to perform miRNA microarray analysis. In plasma, there were 91 differentially expressed miRNAs between HCC and control group (the combination of hepatic cirrhosis and healthy group), which were presented in cluster heatmap (Supplementary Figure S1A) and volcano plot (Supplementary Figure S1B). Meanwhile, there were 203 differentially expressed miRNAs in exosomes (Supplementary Figures S1C,D), indicating the selective enrichment of differential miRNAs in exosomes. Moreover, among the 43 differentially expressed miRNAs contained in both exosomes and plasma, 3 downregulated miRNAs, including miRNA-26a, miRNA-29c, and miRNA-199a, were selected for further studies, which were identified as tumor suppressors in other studies.

### Differential expression and diagnostic performance of the microRNAs in exosomes and plasma

**FIGURE 2:**
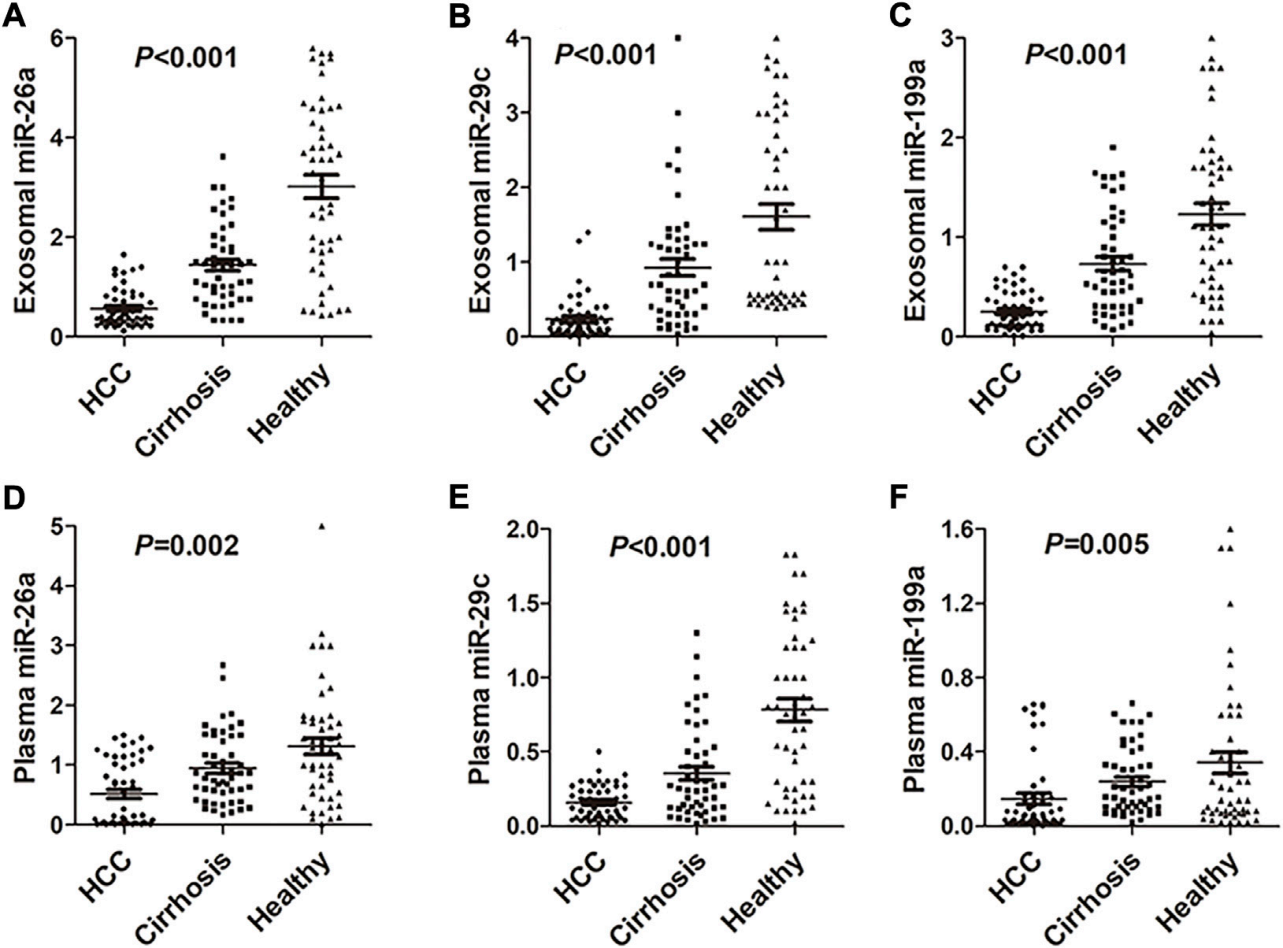
Expression of miRNA-26a, miRNA-29c, and miRNA-199a in HCC, hepatic cirrhosis, and healthy group. **(A–C)** Expression of miRNA-26a **(A)**, miRNA-29c **(B)**, and miRNA-199a **(C)** in exosomes of HCC, hepatic cirrhosis, and healthy group. **(D–F)** Expression of miRNA-26a **(D)**, miRNA-29c **(E)**, and miRNA-199a **(F)** in plasma of HCC, hepatic cirrhosis, and healthy group. One-way analysis was performed among HCC, hepatic cirrhosis, and the healthy group. HCC, hepatocellular carcinoma.

Results of RT-qPCR indicated that the expression of miRNAs (including miRNA-26a, miRNA-29c, and miRNA-199a) in exosomes was significantly lower in HCC patients compared with hepatic cirrhosis and healthy group (*p* < 0.001; Figures 2A–C). Expression of plasma miRNAs was also down-regulated in HCC patients compared with hepatic cirrhosis and healthy group (*p* = 0.002 for miRNA-26a, *p* < 0.001 for miRNA-29c, and *p* = 0.005 for miRNA-199a; Figures 2D–F). The differential expression of exosomal miRNAs was more evident than miRNAs in plasma. Expression of tissue miRNAs was also downregulated in HCC patients compared with the healthy group (*p* < 0.0001 for miRNA-26a, miRNA-29c, and miRNA-199a; Supplementary Figures S2A–C) in TCGA-LIHC cohort. The expression of plasma and exosomal miRNAs in our cohort was consistent with the TCGA-LIHC cohort.

**FIGURE 3:**
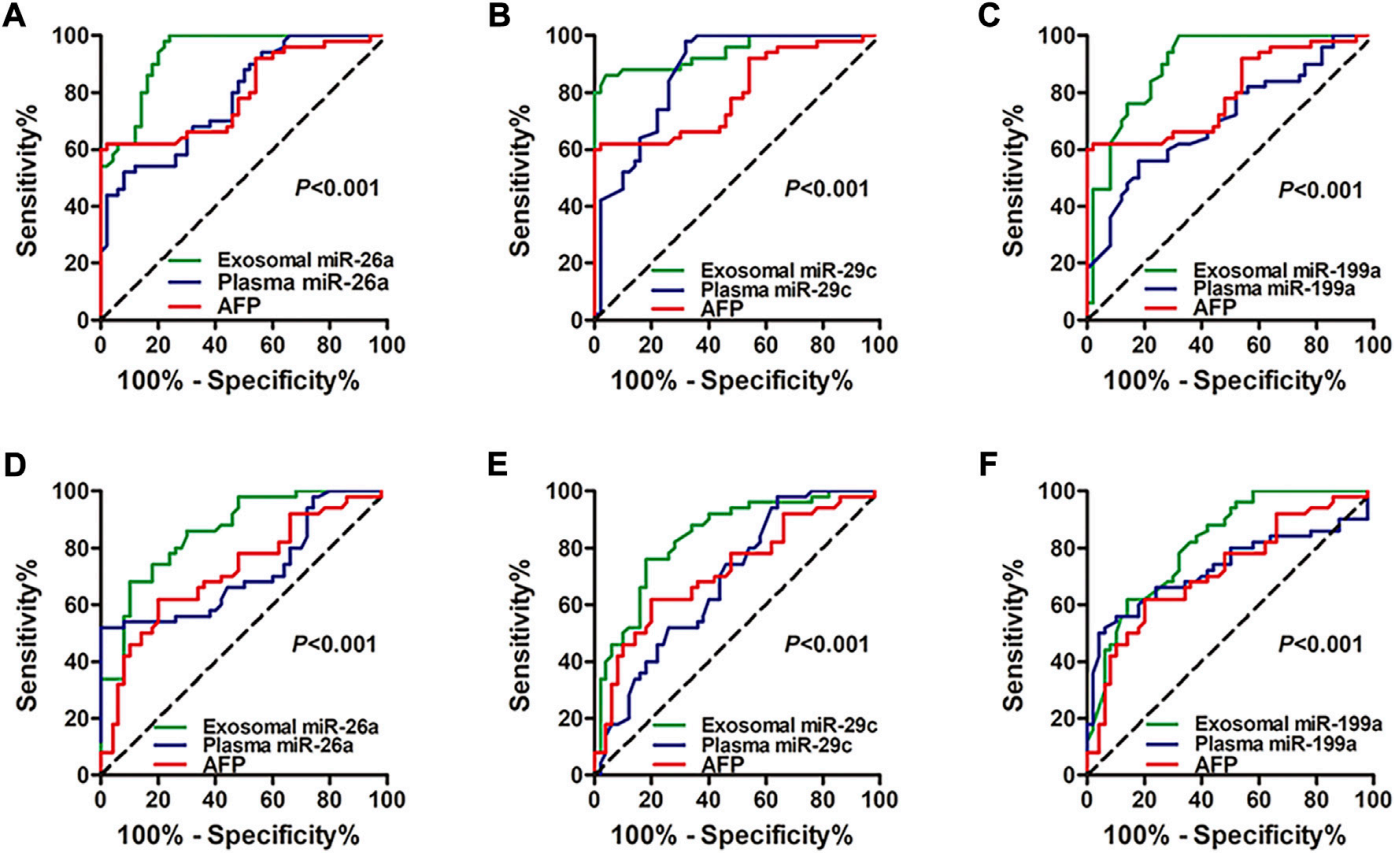
ROC curve analysis shows the diagnostic performance of miRNA-26a, miRNA-29c, and miRNA-199a in exosomes and plasma. **(A–C)** Diagnostic performance of exosomal and plasmic miRNA-26a **(A)**, miRNA-29c **(B)**, and miRNA-199a **(C)** in distinguishing the HCC group from the healthy group. **(D–F)** Diagnostic performance of exosomal and plasmic miRNA-26a **(D)**, miRNA-29c **(E)**, and miRNA-199a **(F)** in distinguishing the HCC group from the hepatic cirrhosis group. Diagnostic ROC curve analysis of AFP was also performed **(A–F)**. The *X*-axis indicated a false positive rate, and *Y*-axis indicated a true positive rate. HCC, hepatocellular carcinoma; ROC curve, receiver operating characteristic curve; AFP, alpha-fetoprotein.

The ROC curve was conducted and the diagnostic performance of the miRNAs was evaluated. Compared with miRNAs and AFP in plasma, exosomal miRNAs showed significantly higher accuracy in discriminating the HCC group from the healthy group (*p* < 0.001 for miRNA-26a, miRNA-29c, and miRNA-199a; Figures 3A–C) and hepatic cirrhosis group (*p* < 0.001 for miRNA-26a, miRNA-29c, and miRNA-199a; Figures 3D–F). The ROC curve and diagnostic performance of tissue miRNAs were also conducted in the TCGA-LIHC cohort. The AUC of miRNA-26a, miRNA-29c and miRNA-199a was 0.8901, 0.9171, and 0.8501, respectively (Supplementary Figures S2D–F). The results showed that exosomal miRNAs of our cohort identified optimal diagnostic performance.

### Kyoto encyclopedia of genesand genomes enrichment analysisof the microRNAs

KEGG pathway analyses were performed to elucidate the pathways that were associated with miRNAs. KEGG analysis results showed that several cancer-related and infection molecular functions were significantly enriched, such as Pathways in cancer, Viral carcinogenesis, Hepatitis B, PI3K-Akt signaling pathway, Focal adhesion, MAPK signaling pathway, MicroRNAs in cancer, FoxO signaling pathway, and HIF-1 signaling pathway (Supplementary Figures S3A–C).

### Establishment and evaluation of the predictive microRNA panel

**FIGURE 4:**
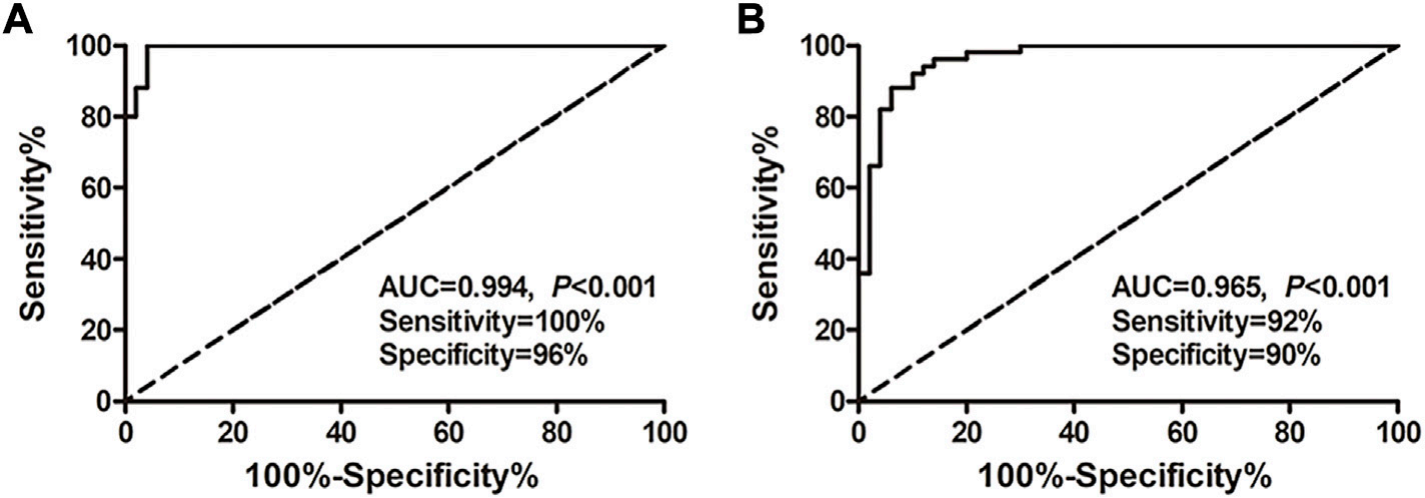
Performance of the exosomal miRNA panel in HCC diagnosis by ROC curve analysis. **(A)** Diagnosis of HCC from the healthy group. **(B)** Diagnosis of HCC from the hepatic cirrhosis group. The *X*-axis indicated a false positive rate, and *Y*-axis indicated a true positive rate. HCC, hepatocellular carcinoma; ROC curve, receiver operating characteristic curve.

Considering the superior diagnostic value of exosomal miRNAs for HCC, multivariate logistic regression analysis on the variables of miRNA-26a, miRNA-29c, and miRNA-199a was performed. And these miRNAs were combined as a panel using the calculation formula as follows: logit (*p* = HCC) = 7.401 – 3.724 × miR26a – 2.894 × miR29c – 7.430 × miR199a. ROC curve was conducted and the performance of the established miRNA panel in differentiating the HCC group from hepatic cirrhosis and the healthy group was evaluated. The results demonstrated that the exosomal miRNA panel had high accuracy in discriminating HCC group from either healthy (sensitivity = 100%, specificity = 96%, AUC = 0.994; Figure 4A) or hepatic cirrhosis group (sensitivity = 92%, specificity = 90%, AUC = 0.965; Figure 4B). We also conducted a ROC curve analysis of the miRNAs panel of the TCGA-LIHC cohort. The results showed that the tissue miRNA panel of the HCC group from the healthy group had relatively low accuracy (sensitivity = 85%, specificity = 96%, AUC = 0.957; Supplementary Figure S2G) compared to the exosomal miRNA panel of our cohort.

## Discussion

In this study, the expression profiles of miRNAs in blood samples derived from HCC patients, hepatic cirrhosis patients, and healthy subjects were detected by miRNA microarray analysis. The candidate miRNAs, including miRNA-26a, miRNA-29c, and miRNA-199a, were selected for further investigation. The expression of miRNAs in exosomes and plasma was detected. In addition, the diagnostic accuracy of miRNAs and exosomal miRNA panel was explored. The results indicated differential expression of candidate miRNAs among HCC, hepatic cirrhosis, and the healthy group, consistent with microarray analysis results. When distinguishing HCC from cirrhosis and healthy groups, exosome miRNA showed perfect diagnostic performance.

Under the influence of factors such as hypoxia and internal environmental changes, tumor cells can secrete a large number of exosomes, which are present in urine, pleuroperitoneal fluid, and peripheral blood. Exosomes have pleiotropic biological functions, including antigen-presenting, intracellular communication, signal transmission, and transferring of nucleic acids and proteins (Zhang et al., 2015). Accumulating evidence has revealed that exosome miRNAs were identified as novel non-invasive biomarkers for various cancer types (Yoshikawa et al., 2018; Chen et al., 2019; Kawamura et al., 2019). And it has been reported that exosomal miRNAs could be used as biomarkers for the diagnosis and prognosis of HCC, such as miRNA-21, miRNA-122, miRNA-148a, and miRNA-1246 (Liese et al., 2016; Pan et al., 2018; Xu et al., 2018).

This study focused on miRNA-26a, miRNA-29c, and miRNA-199a, which were screened by microarray analysis and testified by RT-qPCR. MiRNA-26a locates in a fragile chromosomal region associated with various human cancers and increasing studies have shown that deregulation of its expression occurs in several types of cancer. MiRNA26a participates in various biological pathways, including tumor cell proliferation, invasion, differentiation, angiogenesis, energy metabolism, etc. Moreover, it has been confirmed that many of miRNA targets are oncogenes (Liu et al., 2015; Miyamoto et al., 2016; Chen et al., 2017; Liu et al., 2018). Studies have shown that miRNA-26a not only plays a role in tumorigenesis as a tumor suppressor but also may affect tumor development in lung cancer and glioma as an oncogene (Liu et al., 2012; Qian et al., 2013). Other researchers explored the potential ability of miRNA-26a as a biomarker for human cancers, and it has been reported that miRNA-26a could predict the treatment response of HCC and glioblastoma multiforme (Kim et al., 2018; Sippl et al., 2019). MiRNA-29c has been demonstrated to be down-regulated in several types of cancer by several studies, which acts as a tumor suppressor by inhibiting cell proliferation and migration, as well as reducing the resistance to chemotherapy and radiation therapy (Hudcova et al., 2016; Zhang et al., 2017; Li et al., 2018; Sun et al., 2018). Similarly, miRNA-199a has also been shown to repress the malignant behavior of cancer cells through its downstream genes in breast cancer, hepatocellular carcinoma, lung cancer, prostate cancer, and papillary thyroid carcinoma (Chen et al., 2016; Ahmadi et al., 2017; Qu et al., 2017; Zhan et al., 2017; Ma et al., 2018). Our study revealed that miRNAs (including miRNA-26a, miRNA-29c, and miRNA-199a) in exosomes and plasma were down-regulated in the HCC group compared with hepatic cirrhosis and healthy group, indicating that these miRNAs might be tumor suppressors in HCC, which was in accordance with other researchers. And these miRNAs showed perfect diagnostic performance in distinguishing the HCC group from the hepatic cirrhosis or healthy group. The results show that exosomal miRNAs can more accurately distinguish the HCC group from other groups. The diagnostic performance of exosomal and plasma miRNA in our cohort were much higher than the tisssue miRNAs in TCGA-LIHC cohort. Therefore, an exosomal miRNA panel including miRNA-26a, miRNA-29c, and miRNA-199a was conducted, which showed perfect diagnostic performance and could be a potential biomarker for HCC diagnosis.

At last, there are still some limitations of this study. Firstly, the sample size is relatively small, and further studies with a larger sample size are needed to confirm the present results. Secondly, the performance of miRNAs and miRNA panel in predicting the prognosis of HCC should be evaluated in further studies.

## Conclusion

In this study, we proved that exosomal miRNA panel, including miRNA-26a, miRNA-29c, and miRNA-199a, might be a reliable biomarker for diagnosing HCC. And as a non-invasive approach, gene detection with exosomes would have broad application prospects in the diagnosis and treatment of tumors.

## Data Availability

The data of 150 samples for this study are included in the article/Supplementary Material. The LIHC dataset was collected from The Cancer Genome Atlas (TCGA) (http://xena.ucsc.edu/).
