# Supplementary material for: Exosomal microRNA panel as a diagnostic biomarker in patients with hepatocellular carcinoma
Source: Front Cell Dev Biol. 2022 Sep 23;10:927251. doi: 10.3389/fcell.2022.927251 (PMC9537616; doi:10.3389/fcell.2022.927251)
Supplement: Supplementary file 3 [file DataSheet1.docx]

Supplementary Material

## Supplementary Figures


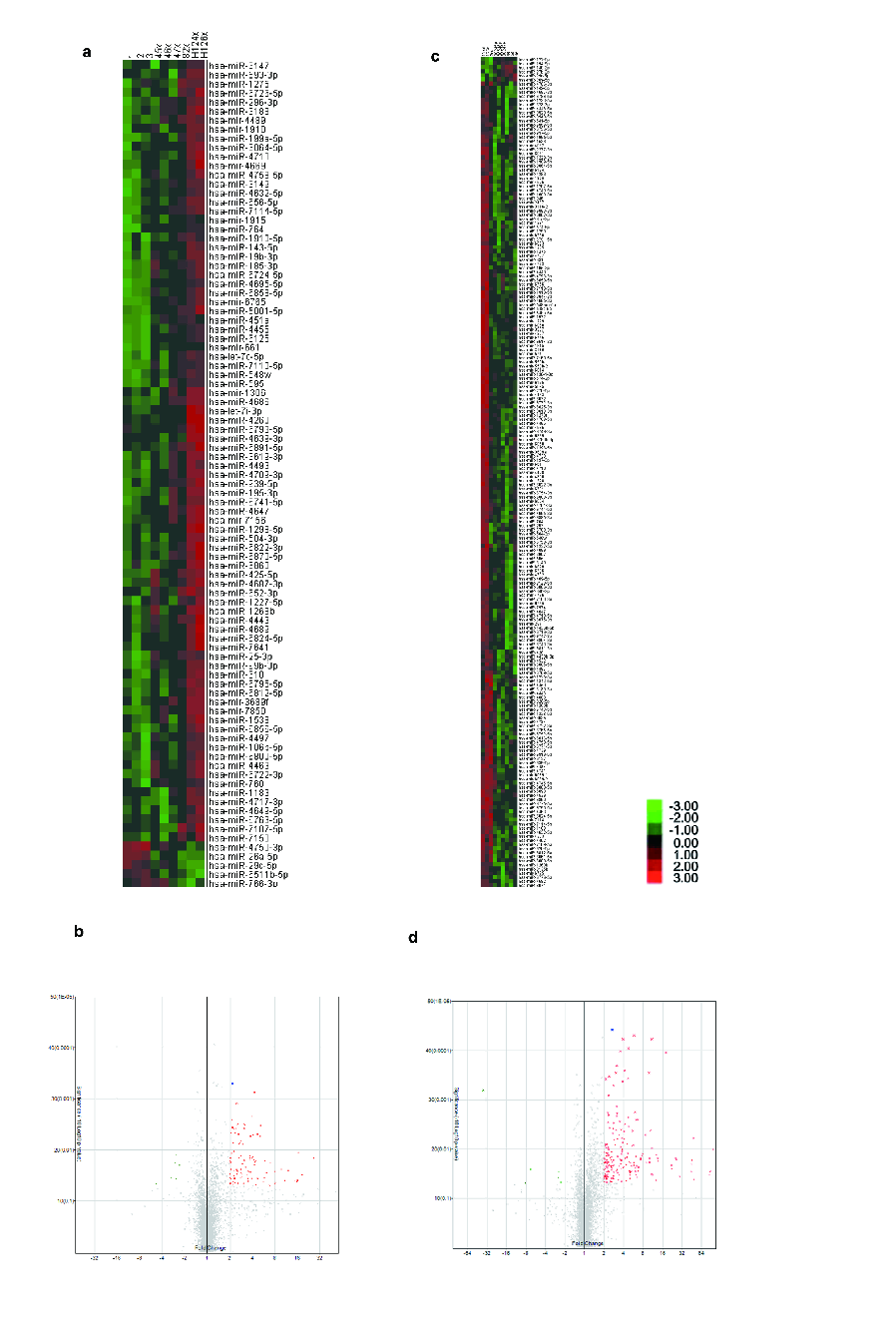


**Supplementary Figure 1.** Analysis of differentially expressed miRNAs in exosomes and plasma by miRNA microarray. (a and c) Cluster heatmap of differentially expressed miRNAs in plasma (a) and exosomes (c). Each row represented a miRNA and each column represented a sample. Red indicated up-regulation and green indicated down-regulation. (b and d) Differentially expressed miRNAs in plasma (b) and exosomes (d) were shown in volcanic plots. Up-regulated miRNAs with fold change ≥ 2 and *P*<0.05 were shown in red. Down-regulated miRNAs with fold change ≤ -2 and *P*<0.05 were shown in green. The comparison was carried out between HCC group and control group (the combination of hepatic cirrhosis and healthy groups).


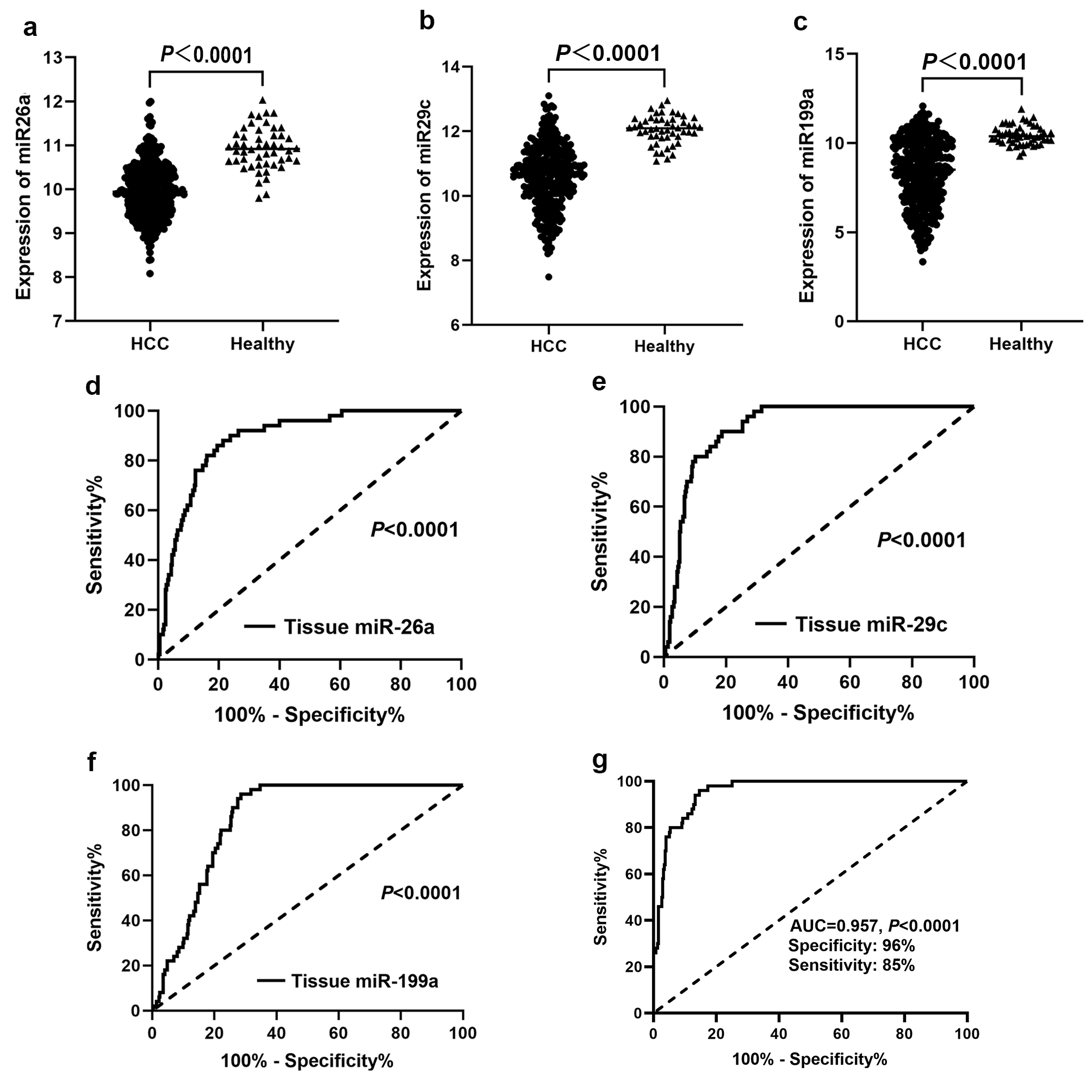


**Supplementary Figure 2.** Three miRNAs analysis in TCGA-LIHC. (a-c) Expression of miRNA-26a (a), miRNA-29c (b) and miRNA-199a (c) in tissue of HCC and healthy group in TCGA-LIHC cohort. (d-f) Diagnostic performance of exosomal and plasmic miRNA-26a (d), miRNA-29c (e), miRNA-199a (f) in distinguishing HCC group from healthy group in TCGA-LIHC cohort. (g) Performance of the tissue miRNA panel in HCC diagnosis in TCGA-LIHC cohort.


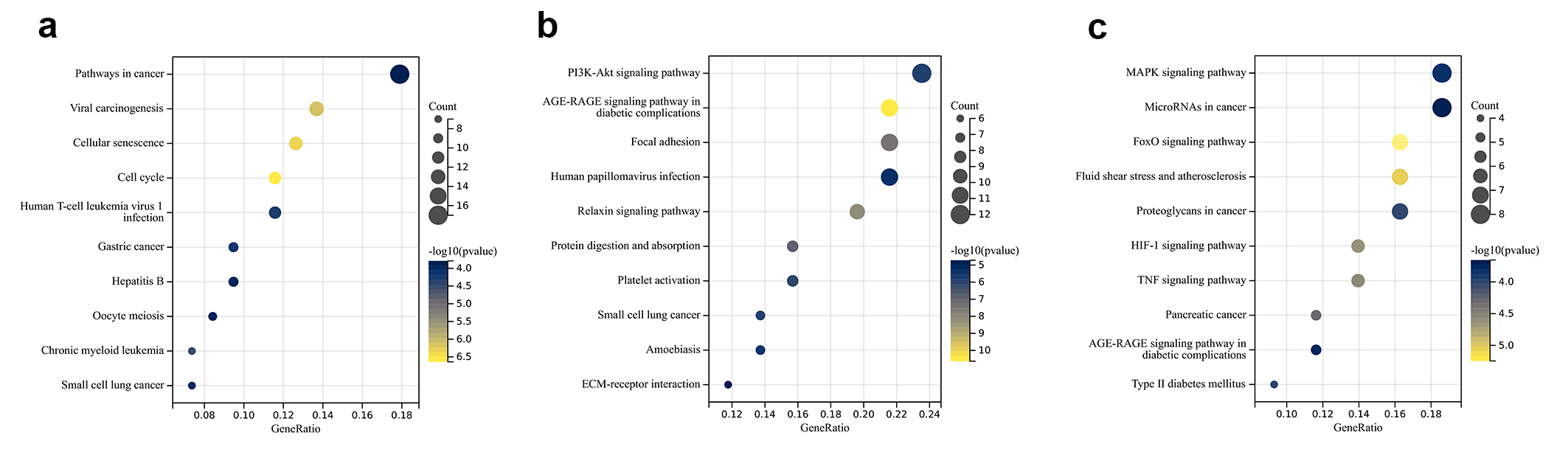


**Supplementary Figure 3.** Functional enrichment analysis for three miRNAs. (a-c) The results of KEGG pathways analysis of miRNA-26a targets(a), miRNA-29c targets(b), and miRNA-199a targets(c).
